# Preliminary safety assessment of oridonin in zebrafish

**DOI:** 10.1080/13880209.2019.1662457

**Published:** 2019-09-23

**Authors:** Lili Tian, Donglai Sheng, Qiushuang Li, Chenxu Guo, Guofu Zhu

**Affiliations:** a School of Pharmacy, Shanghai University of Traditional Chinese Medicine, Shanghai, China;; b Traditional Chinese Medicine Pharmacy, Zhejiang Hospital, Hangzhou, China;; c Institute of Developmental and Regenerative Biology, Hangzhou Normal University, Hangzhou, China;; d Center of Clinical Evaluation and Analysis, Zhejiang Provincial Hospital of Traditional Chinese Medicine, Hangzhou, China;; e Department of Integrated Chinese and Western Medicine, Shanghai Eastern Hepatobiliary Surgery Hospital, Shanghai, China

**Keywords:** *Isodon rubescens*, medicinal plant, embryonic development effects, body length, swimming speed, VEGFR3

## Abstract

**Context:** Oridonin, isolated from the leaves of *Isodon rubescens* (Hemsl.) H.Hara (Lamiaceae), has good antitumor activity. However, its safety *in vivo* is still unclear.

**Objective:** To investigate the preliminary safety of oridonin in zebrafish.

**Materials and methods:** Embryo, larvae and adult zebrafish (*n* = 40) were used. Low, medium and high oridonin concentrations (100, 200 and 400 mg/L for embryo; 150, 300 and 600 mg/L for larvae; 200, 400 and 800 mg/L for adult zebrafish) and blank samples were administered. At specific stages of zebrafish development, spontaneous movement, heartbeat, hatching rate, etc., were recorded to assess the developmental effects of oridonin. *VEGFA*, *VEGFR2* and *VEGFR3* gene expression were also examined.

**Results:** Low-dose oridonin increased spontaneous movement and hatching rate with median effective doses (ED_50_) of 115.17 mg/L at 24 h post-fertilization (hpf) and 188.59 mg/L at 54 hpf, but these values decreased at high doses with half maximal inhibitory concentrations (IC_50_) of 209.11 and 607.84 mg/L. Oridonin decreased heartbeat with IC_50_ of 285.76 mg/L at 48 hpf, and induced malformation at 120 hpf with half maximal effective concentration (EC_50_) of 411.94 mg/L. Oridonin also decreased body length with IC_50_ of 324.78 mg/L at 144 hpf, and increased swimming speed with ED_50_ of 190.98 mg/L at 120 hpf. The effects of oridonin on zebrafish embryo development may be attributed to the downregulation of *VEGFR3* gene expression.

**Discussions and conclusions:** Oridonin showed adverse effects at early stages of zebrafish development. We will perform additional studies on mechanism of oridonin based on VEGFR3.

## Introduction

Angiogenesis, the endothelium-derived development of new blood vessels, plays a crucial role in biological and pathological processes. Specific antiangiogenic agents are an attractive therapeutic approach in treating angiogenesis-dependent diseases, particularly in solid tumour progression and diabetic retinopathy (Carmeliet and Jain 2011). Vascular endothelial growth factor (VEGF) is one of the most important and specific angiogenic factors that regulate normal physiological and pathological neovascularization. VEGFA is the main factor promoting angiogenesis. VEGFR2 and VEGFR3 are important receptors that mediate the angiogenic activity of VEGF through distinct signal transduction pathways that regulate endothelial cell proliferation, migration, differentiation and tube formation (Shibuya 2011).

However, generally available antiangiogenic agents have serious side effects, such as hypertension, bleeding and gastrointestinal perforation, which limit their use (Zuo et al. 2014; Frandsen et al. 2016). To discover safe and efficient agents for treating angiogenesis-related diseases, researchers have focused on developing small-molecule therapeutic strategies that target the VEGF signalling pathway (Siveen et al. 2017).

Based on extensive research, traditional Chinese medicine reveals an improved curative effect and reduced side effects, compared with clinical therapy (Lu et al. 2016; Kumar et al. 2016; Choi et al. 2016; Park et al. 2017). Oridonin, an effective diterpenoid compound isolated from the traditional Chinese herb *Isodon rubescens* (Hemsl.) H.Hara (Lamiaceae), has antibacterial, anti-inflammatory, proapoptotic, antitumor and other pharmacological properties (Zhou et al. 2007; Kang et al. 2010a, 2010b; Gao et al. 2010; Liu et al. 2016; Cao et al. 2016). Notably, the underlying mechanism of oridonin in inhibiting tumour growth and metastasis is through its antiangiogenic activity by blocking c-Met, Notch and VEGF signalling (Meade-Tollin et al. 2004; Dong et al. 2014; Liu et al. 2014; Xia et al. 2017; Tian et al. 2017). Oridonin has been shown to exert anticancer effects and bypass major drug resistance mechanisms, which might be effective against drug refractory tumours (Kadioglu et al. 2018).

Our previous research showed that oridonin inhibits tumour growth and metastasis *in vitro* and *in vivo*, which may be attributed to its antiangiogenic effects (Tian et al. 2017). However, the safety of oridonin *in vivo* is still unclear.

In recent years, zebrafish is increasingly used as an alternative model organism for studying a wide range of biological phenomena and mechanisms owing to its prolific nature, relative ease of maintenance and sufficiently high genetic homology to humans (Tsang et al. 2017). This is especially true for the embryonic zebrafish model, which offers whole-animal investigations with convenience, such as reductions in animal usage, experimental time and costs, and quantity of the tested compound. In addition, the majority of morphological and developmental changes in this model can be evaluated noninvasively because of the transparent nature of the embryos (Truong and Tanguay 2017).

In the present study, we selected embryo, larvae and adult zebrafish models to investigate the safety of oridonin and evaluate the effects of oridonin on zebrafish development by assessing different end-points and exposure periods. Moreover, this study elucidated the mechanism of the effects of oridonin on the expression *VEGFA*, *VEGFR2* and *VEGFR3*, key genes in the VEGF signalling pathway.

## Materials and methods

### Compound and reagents

Oridonin (No: 1126YA13, purity more than 98%) was purchased from Shanghai Yuanye Bio-Technology Co. Ltd., China. Oridonin stock solution (100 g/L) was diluted in 100% dimethyl sulfoxide (DMSO) and stored at 4 °C in the dark. Next, the solution was serially diluted in 100% DMSO that was 1000 times more concentrated to allow for a 1:1000 dilution with embryo medium (EM) (0.137 M NaCl, 5.4 mM KCl, 0.25 mM Na_2_HPO_4_, 0.44 mM KH_2_PO_4_, 1.3 mM CaCl_2_, 1.0 mM MgSO_4_ and 4.2 mM NaHCO_3_) (Westerfield 2000) to create a serial dilution with a final DMSO concentration of 1%. DMSO and tricaine methanesulfonate (MS-222) were obtained from Sigma. Trizol (No: 11667165001) was purchased from Roche. Recombinant RNase (No: 2313 A), Reverse TranScriptase M-MLV (No: 2641 A) and SYBR Premix Ex TaqTM (No: RR420) were purchased from Takara. The other chemicals used in this study were of analytical grade.

### Fish husbandry and embryo collection

All study protocols involving zebrafish were approved by the Animal Care and Use Committee of Shanghai University of Traditional Chinese Medicine. Forty zebrafish (male and female, 1:1) were obtained from the Institute of Biochemistry and Cell Biology, Shanghai Institutes for Biological Sciences, Chinese Academy of Sciences. All fishes were housed in our laboratory and raised under standard laboratory conditions. All fishes were at the juvenile stage, cultured in our laboratory until sexual maturation for spawning, and then raised and maintained at standard laboratory conditions of 28 °C under a 14/10 h light/dark photoperiod in a recirculation system, according to the standard zebrafish breeding protocols (Westerfield 2000). The water supplied to the system was filtered using reverse osmosis (pH 7.0–7.5), and Instant Ocean^®^ salt was added to this water to increase its conductivity to 450–1000 μS/cm (system water). The fishes were fed live *Artemia* (Jiahong Feed Co., Tianjin, China) twice per day.

To obtain embryos, adult fishes were placed in tanks at a female-to-male ratio of 1:1, and spawning was induced in the morning when the light was turned on. Embryos were collected within 0.5 h of spawning, rinsed in EM, and then incubated in Petri dishes at 28 ± 1 °C until chemical treatment. Fertilized embryos with normal morphology were staged under a dissecting microscope (SMZ 1500, Nikon, Japan) according to standard methods (Kimmel et al. 1995).

### Zebrafish embryo assay

Healthy and synchronously hatched zebrafish embryos (*n* = 40) were selected at 6 h post fertilization (hpf) and treated with EM or with different concentrations of oridonin (100, 200 and 400 mg/L) in 96-well plates (one embryo per well, with 200 μL of solution) for continuous development until 144 hpf. Embryos development in each well was observed using an inverted dissecting microscope (Leica Microsystems, Wetzlar, Germany) at certain observation times. The endpoints include spontaneous movement, heartbeat, hatching rate, nonlethal malformation, swimming speed, and body length and weight, were selected for assessing the effect of oridonin. Embryos were considered dead when they have no heartbeat and dead embryos were immediately removed at each observation time.

Spontaneous movements of the tails of embryos treated with different concentrations of oridonin (100, 200 and 400 mg/L) were counted for 20 s at 24, 27 and 30 hpf. Heartbeat was counted for 10 s at 48, 51 and 54 hpf. Spontaneous movement and heartbeats were recorded after adapting the embryos to room temperature for 15 min. The total recording duration from the first to the last well was <30 min. The assay was repeated independently at least three times, and 10 embryos per group were used each time.

Hatching of the embryos treated with different concentrations of oridonin (100, 200 and 400 mg/L) was recorded from 48 hpf. Successfully hatched embryos were counted until 72 hpf to determine hatching rate. The assay was repeated independently at least three times and 10 embryos were used per group each time.

Occurrence of nonlethal malformation was recorded using a microscope from 6 to 120 hpf, and the EC_50_ of oridonin was determined based on the occurrence of nonlethal malformation. The assay was repeated independently at least three times and 10 embryos were used per group each time.

Swimming speed was determined using a VideoTrack for Zebrafish^TM^ system (Viewpoint, France). The number of movements, distances travelled and total durations of movements were recorded every 60 s and analyzed. The settings of the VideoTrack system were as follows: detection threshold, 18; duration time, 20 min; movement threshold, inact/small, 3.0 mm/s and small/large, 6.0 mm/s; light sequence, duration, 20 min. At 84 hpf, larvae were individually loaded into 24-well plates, with each well containing 2 mL of EM and then observed until 120 hpf. The larvae swimming speed was tested by the VideoTrack for Zebrafish™ system. The assay was repeated independently at least three times and 10 embryos were used per group each time.

Body length and weight were measured at 144 hpf. Larvae were anesthetized by MS-222 and then weighed; images were captured using a microscope. The assay was repeated independently at least three times and 10 embryos were used per group each time.

### Larval zebrafish assay

Healthy larvae zebrafish were selected at 48 hpf and treated with EM or with different concentrations of oridonin (150, 300 and 600 mg/L) in 96-well plates (one larva per well, with 200 μL of solution) for continuous development until 144 hpf. Occurrence of nonlethal malformation was recorded from 48 to 120 hpf, and the EC_50_ of oridonin with respect to nonlethal malformation was determined. Nonlethal malformations were imaged using a microscope. The assay was repeated independently at least three times and 10 larvae were used per group each time.

Swimming speed was measured using the VideoTrack for Zebrafish™ system. The number of movements, distances travelled and total durations of movements were collected every 60 s and analyzed. The settings of the VideoTrack system were as follows: detection threshold, 18; duration time, 20 min; movement threshold, inact/small, 3.0 mm/s and small/large, 6.0 mm/s; light sequence, duration, 20 min. At 84 hpf, larvae were individually loaded into 24-well plates, with each well containing 2 mL of EM, and observed until 120 hpf. The larvae swimming speed was tested using the Video Track for Zebrafish™ system. The assay was repeated independently at least three times and 10 larvae were used per group each time.

Body length and weight were measured at 144 hpf. Larvae were anesthetized by MS-222 and then weighed; images were captured using a microscope. The assay was repeated independently at least three times and 10 larvae were used per group each time.

### Adult zebrafish assay

Healthy adult zebrafish were selected at 3 months post-fertilization (mpf) and treated with EM or with different concentrations of oridonin (200, 400 and 800 mg/L) in tanks (10 zebrafish per tank, with 10 L of solution) for continuous development until 5 mpf.

Sperm vitality was tested in adult male zebrafish. At 5 mpf, four male fish from each group were selected and anesthetized by MS-222. Total body length and wet weight were measured. Next, the testis was surgically removed and weighed. Sperm vitality was determined by a computer-assisted sperm analysis system, according to a previously published procedure (Jing et al. 2009).

Survival rate of fertilized eggs was tested at 5 mpf using six zebrafish per tank from each group. Embryos were obtained from adult zebrafish in tanks with a female-to-male sex ratio of 1:1, and spawning was induced in the morning when the light was turned on. Embryos were collected within 0.5 h of spawning and rinsed in EM. Fertilized embryos with normal morphology were staged under a dissecting microscope (SMZ 1500; Nikon, Japan) according to the standard methods (Kimmel et al. 1995). Survival rate was calculated from 0.5 to 6 hpf.

Hatching rate was recorded from 48 hpf, and successfully hatched embryos were counted until 60 hpf to determine the hatching rate. The assay was repeated independently at least three times and 10 embryos were used per group each time. Occurrence of malformation in the offspring was recorded from 6 to 72 hpf. Nonlethal malformations were imaged using a microscope. The assay was repeated independently at least three times and 10 embryos were used per group each time.

The swimming speed of the offspring was measured using the VideoTrack for Zebrafish^TM^ system. The assay was repeated independently at least three times and 10 larvae were used per group each time.

The body length and weight of the offspring were measured at 144 hpf. Larvae were anesthetized by MS-222 and then weighed; images were captured using a microscope. The assay was repeated independently at least three times and 10 larvae were used per group each time.

### RNA isolation and reverse transcription real-time PCR (qRT-PCR)

RNA samples were obtained from zebrafish embryos, larval zebrafish and the adult zebrafish. For zebrafish embryos, *VEGFA, VEGFR2* and *VEGFR3* gene expression were examined in the control and the oridonin groups (100, 200 and 400 mg/L). A total of 10 larvae per group were homogenized in 1 mL of Trizol reagent (Invitrogen, Carlsbad, CA) with a homogenizer (Polytron, Kinematica, Littau, Switzerland), and total RNA was extracted from the homogenate according to the manufacturer’s protocol. The OD260/OD280 ratio and banding patterns were routinely checked on a 1% agarose gel to ensure the purity and integrity of the RNA sample. Reverse transcription was conducted using an M-MLV reverse transcriptase kit (Takara Biochemicals, Dalian, China) according to the manufacturer’s protocol. For qPCR, 2 μL of the reverse transcription product was amplified directly on a 7300 Real-Time PCR System (Applied Biosystems, Foster City, CA) using 20 μL of SYBR reaction solution. *VEGFA, VEGFR2* and *VEGFR3* gene expression was also examined in the control and oridonin groups (150, 300 and 600 mg/L) of larval zebrafish, as well as in the control and oridonin groups (200, 400 and 800 mg/L) of adult zebrafish. The primers (Biotechnology, Shanghai, China) were designed according to the cDNA sequences from the NCBI database (Table 1).

**Table 1.** Sequences of primers used in the reverse transcription polymerase chain reaction.

Gene name	Gene bank ID	Sequence of primers (Forward)	Sequence of primers (Reverse)
VEGFA	30682	CAGCTGTCAAGAGTGCCTACATAC	CATCAGGGTACTCCTGCTGAATTTC
VEGFR2	796537	TCACATGGTTTGGTAGAGGGATCTC	GTGCAGTTGATCCTCTGCAAATGAG
VEGFR3	30121	TCTGTCGGATTTGGATTGGGA	TTGGTGTTGTCAAGGGTGGG
β-Actin	57934	CGAGCAGGAGATGGGAACC	CAACGGAAACGCTCATTGC

### Statistical analysis

All data were reported as mean ± standard error of the mean (SEM). The *t*-test for independent analysis was applied to evaluate the differences between the treatment and control groups, and a value of *p* < 0.05 was considered statistically significant.

## Results

### Effect of oridonin on the spontaneous movements of zebrafish embryos

**Figure 1.**
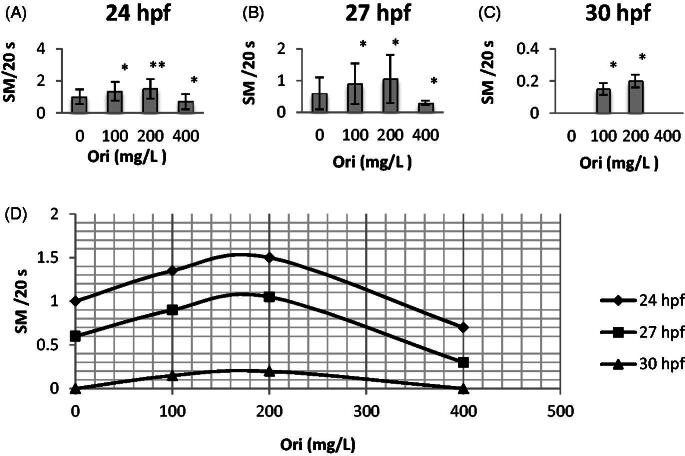
Effects of oridonin on spontaneous movements for zebrafish embryos. Note: Asterisks indicate statistically significant differences between different concentration of oridonin groups and the control (without oridonin) group (*p < 0.05; **p < 0.01). Each error bar represents the standard deviations of at least three experiments. A: 24 hpf; B: 27 hpf; C: 30 hpf. SM: Spontaneous Movements; Ori: Oridonin.

The first observed effect of oridonin on zebrafish embryo development was the influence on the spontaneous movement frequency from 24 to 30 hpf. Embryos in the control group (without oridonin) showed mean spontaneous movements of 1.00 at 24 hpf, 0.60 at 27 hpf, and 0.00 at 30 hpf; however, these movements decreased in the oridonin groups (Figure 1(D)).

After exposure to oridonin until 30 hpf, the number of spontaneous movements of embryos first increased and then decreased (Figure 1(A–C)). At 24 hpf, the number of spontaneous movements was the highest in relation to the increasing oridonin concentration; spontaneous movements increased at first and decreased afterwards, with mean values of 1.35, 1.50 and 0.70 for the 100, 200 and 400 mg/L groups, respectively (Figure 1(A)), and there were significant differences from those of the control group (*p* < 0.05, *p* < 0.01 and *p* < 0.05, respectively). Low-dose oridonin increased spontaneous movement with median effective doses (ED_50_) of 115.17 mg/L at 24 hpf, but the value decreased at high doses with half maximal inhibitory concentration (IC_50_) of 209.11 mg/L.

### Effect of oridonin on the number of heartbeats of zebrafish embryos

**Figure 2.**
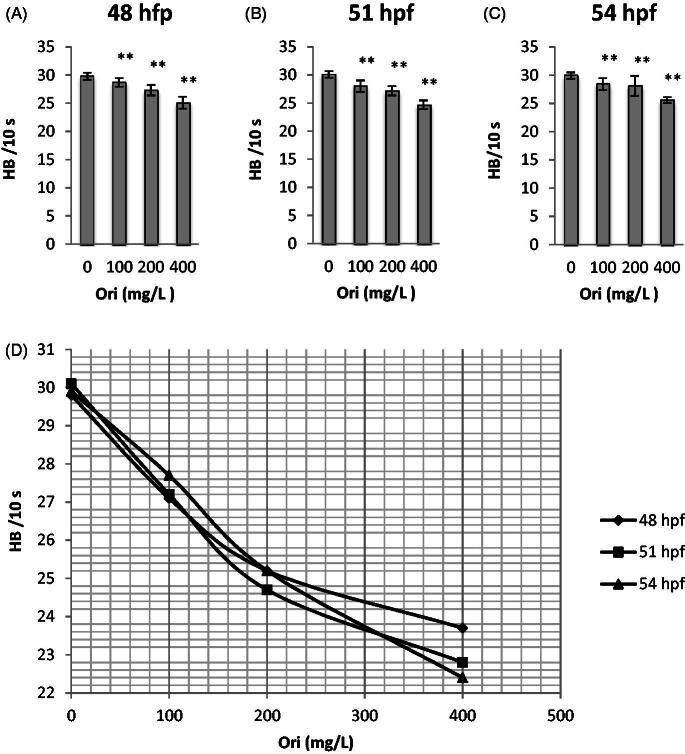
Effects of oridonin on heat beats for zebrafish embryos. Note: Asterisks indicate statistically significant differences between different concentration of oridonin groups and the without oridonin group (**p < 0.01). Each error bar represents the standard deviations of at least three experiments. A: 48 hpf; B: 51 hpf; C: 54 hpf. HB: Heart Beats; Ori: Oridonin.

Oridonin exposure at 48 hpf, which is the early developmental stage of zebrafish, decreased heartbeat frequency. Exposed to oridonin until 54 hpf decreased heartbeat in zebrafish embryos (Figures 2(A–C)). All oridonin groups exhibited significant differences compared with the control (*p* < 0.01).

The embryos in the control group (without oridonin) showed a mean heartbeat number of 29.80 at 48 hpf, 30.10 at 51 hpf, and 29.90 at 54 hpf, whereas the oridonin groups showed steady heartbeats (Figure 2(D)). Oridonin decreased heartbeat with IC_50_ of 285.76 mg/L at 48 hpf.

### Effect of oridonin on the hatching rate of zebrafish embryos

**Figure 3.**
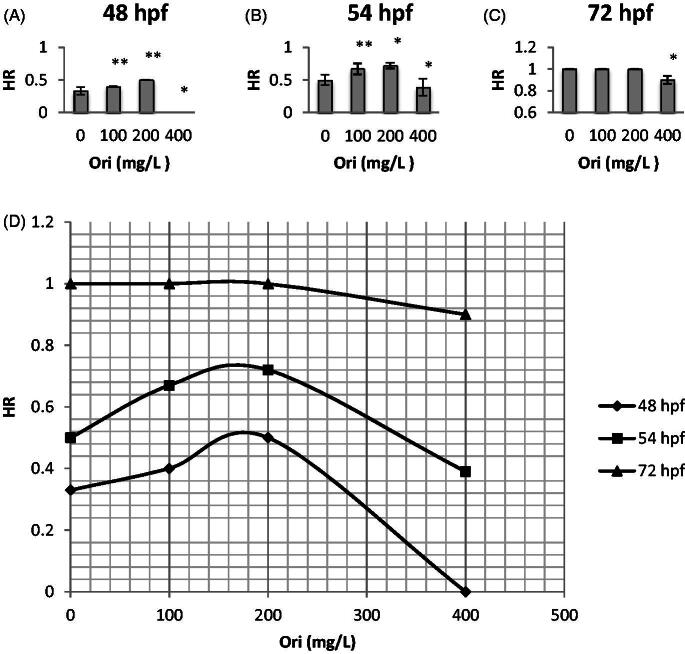
Effects of oridonin on hatching rate for zebrafish embryos. Note: Asterisks indicate statistically significant differences between different concentration of oridonin groups and the without oridonin group (*p < 0.05; **p < 0.01). Each error bar represents the standard deviations of at least three experiments. A: 48 hpf; B: 54 hpf; C: 72 hpf. HR: Hatching Rate; Ori: Oridonin.

Oridonin affected the development of zebrafish embryos from 48 to 72 hpf. Embryos in the control group (without oridonin) showed a mean hatching rate of 0.33 at 48 hpf, 0.50 at 54 hpf, and 1.00 at 72 hpf, whereas the oridonin groups showed increased hatching rate (Figure 3(D)).

On the contrary, the hatching rate of embryos exposed to oridonin until 72 hpf first increased and then decreased (Figure 3(A–C)). At 54 hpf, the number of hatching events was most distinct in relation to the increasing oridonin concentration; the hatching rate increased at first and decreased afterwards, with mean values of 0.67, 0.72 and 0.39 for the 100, 200 and 400 mg/L group, respectively (Figure 3(C)), showing significant differences compared to those of the control (*p* < 0.01, *p* < 0.05 and *p* < 0.05, respectively). Low-dose oridonin increased hatching rate with ED_50_ of 188.59 mg/L at 54 hpf, but the value decreased at high doses with IC_50_ of 607.84 mg/L.

### Effect of oridonin on malformation in zebrafish embryo-larvae

**Figure 4.**
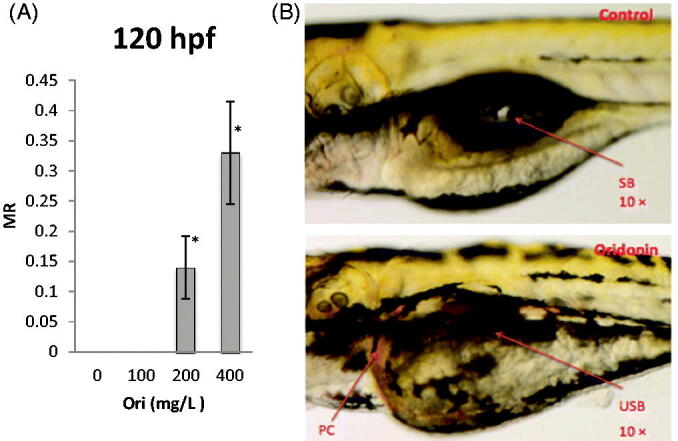
Effects of oridonin on malformation for zebrafish embryo-larvae. Note: Asterisks indicate statistically significant differences between different concentration of oridonin groups and the without oridonin group (*p < 0.05). Each error bar represents the standard deviations of at least three experiments. MR: Malformation Rate; Ori: Oridonin; PC: Pericardial Congestion; USB: Uninflated Swim Bladder; SB: Swim Bladder.

Embryos were exposed to various concentrations of oridonin from 6 to 120 hpf and were monitored daily for mortality until 120 hpf. The control substance (1‰ DMSO) was not toxic to the embryos. The main observed malformations were uninflated swim bladder and pericardial congestion (Figure 4(B)), which were observed in embryos and treated with increasing concentrations of oridonin, at an EC_50_ of 411.94 mg/L. At 120 hpf, the mean malformation rates were 0.14 and 0.33 for the 200 and 400 mg/L oridonin groups, respectively (Figure 4(A)), showing significant differences compared to those of the control (*p* < 0.05 and *p* < 0.05). Oridonin induced malformation at 120 hpf with half maximal effective concentration (EC_50_) of 411.94 mg/L.

### Effect of oridonin on the swimming speed of zebrafish embryo-larvae

**Figure 5.**
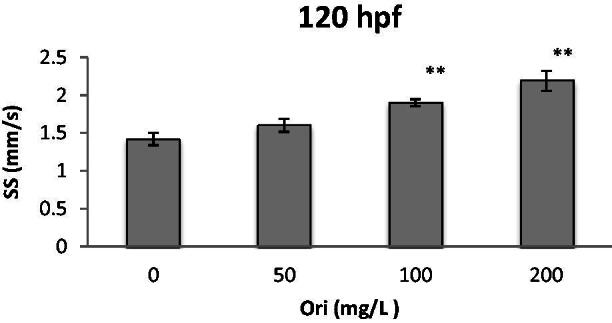
Effects of oridonin on swimming speed for zebrafish embryo-larvae. Note: Asterisks indicate statistically significant differences between different concentration of oridonin groups and the without oridonin group (**p < .01). Each error bar represents the standard deviations of at least three experiments. SS: Swimming Speed; Ori: Oridonin.

The swimming behaviours of zebrafish larvae were examined at 120 hpf. At this period, constant swimming activity with low variation in locomotor activities was observed. The average swimming speed in the control group was 1.42; however, the 100 and 200 mg/L oridonin groups showed significantly (*p* < 0.01 and *p* < 0.01, respectively) increased larvae swimming speed 1.90 and 2.19, respectively (Figure 5). Oridonin increased swimming speed with ED_50_ of 190.98 mg/L at 120 hpf.

### Effect of oridonin on the body length and body weight of zebrafish embryo-larvae

**Figure 6.**
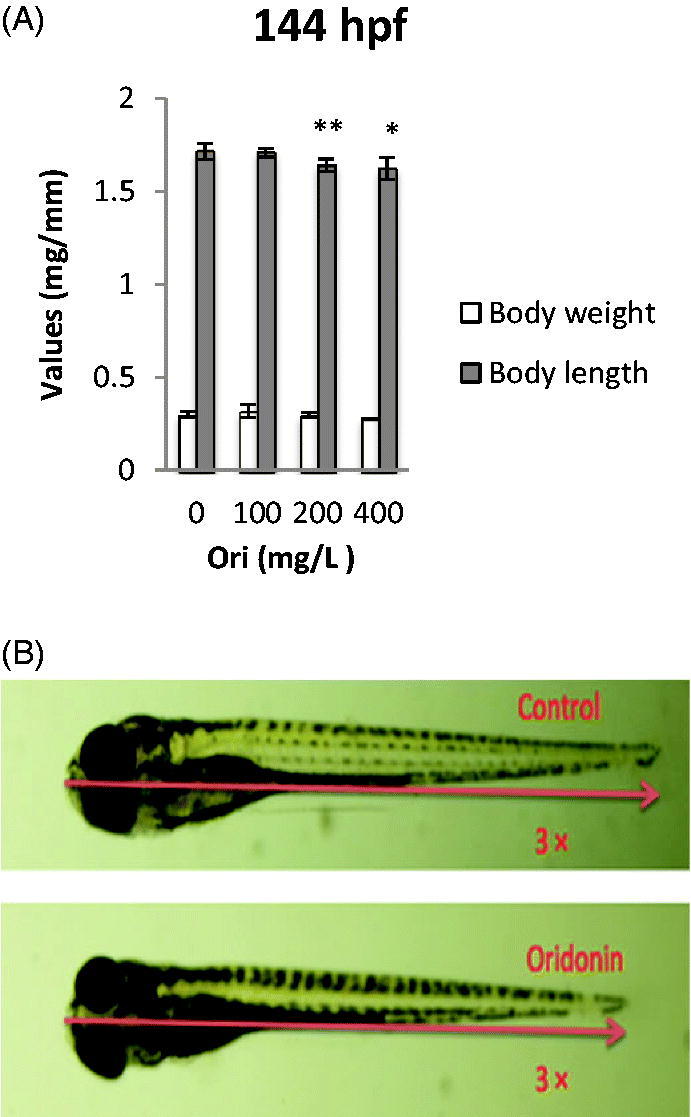
Effects of oridonin on body weight and length for zebrafish embryos. Note: Asterisks indicate statistically significant differences between different concentration of oridonin groups and the without oridonin group (*p < 0.05; **p < 0.01). Each error bar represents the standard deviations of at least three experiments. Ori: Oridonin.

Embryos were exposed to various concentrations of oridonin from 6 to 144 hpf. The body length and weight of the larvae were measured to assess the effects of oridonin at 144 hpf (Figure 6(B)). Exposure of embryos to 100, 200 and 400 mg/L oridonin resulted in larvae body weight of 0.32, 0.30 and 0.28 mg, respectively, compared with 0.30 mg in the control group (Figure 6(A)); the larvae body length was 1.64 and 1.62 mm, respectively, which were significantly different from the 1.72 mm in the control group (*p* < 0.01 and *p* < 0.05, respectively). The embryos exposed to oridonin showed no significant differences in body length compared with that of the control group. Oridonin decreased body length with IC_50_ of 324.78 mg/L at 144 hpf.

### Effect of oridonin on the larvae model

Oridonin exposure did not result in malformation in the larvae model (Table 2). The larvae exposed to oridonin exhibited no significant differences in swimming speed, compared with that of the control group (Table 2). Neither body length nor weight differed significantly between any of the oridonin groups and their respective controls (Table 2).

**Table 2.** Comparison of experiment groups for the larvae zebrafish.

Groups (mg/L)	Malformation rate (%)	Swimming speed (mm/s)	Body length (mm)	Body weight (mg)
0	0	1.58 ± 0.20	0.71 ± 0.030	0.48 ± 0.015
150	0	1.51 ± 0.13	0.72 ± 0.16	0.52 ± 0.035
300	0	1.56 ± 0.21	0.74 ± 0.14	0.55 ± 0.068
600	0	1.50 ± 0.28	0.71 ± 0.11	0.57 ± 0.070

### Effect of oridonin on the adult model

In the adult models, oridonin did not affect sperm vitality, survival rate of fertilized eggs and hatching rate (Table 3). Furthermore, the offspring showed no malformation (Table 3). The swimming speed, body length and body weight did not differ significantly between the oridonin groups and their respective controls (Table 3).

**Table 3.** Comparison of experiment groups for the adult zebrafish and offspring.

Groups (mg/L)	Sperm vitality (%)	Survival rate of fertilized egg (%)	Hatching rate (%)	Malformation rate (%)	Swimming speed (mm/s)	Body length (mm)	Body weight (mg)
0	88.33 ± 0.58	93.33 ± 1.00	97.78 ± 0.58	0	1.95 ± 0.53	0.59 ± 0.0089	0.31 ± 0.021
200	87.00 ± 1.00	94.44 ± 0.58	95.67 ± 0.00	0	2.04 ± 0.35	0.60 ± 0.0045	0.32 ± 0.035
400	87.67 ± 1.53	95.56 ± 0.57	95.55 ± 1.15	0	1.95 ± 0.72	0.59 ± 0.011	0.31 ± 0.015
800	88.00 ± 1.73	91.11 ± 1.53	94.44 ± 0.58	0	2.00 ± 0.41	0.59 ± 0.028	0.32 ± 0.026

### *Effect of oridonin on* VEGFA, VEGFR2 *and* VEGFR3 *gene expressions in zebrafish*

The molecular pathway responsible for the effect of on the zebrafish VEGF pathway was studied *in vivo* by qRT-PCR. VEGFA, VEGFR2 and VEGFR3 are important molecules that enhance zebrafish development, especially during the embryo stage.

**Figure 7.**
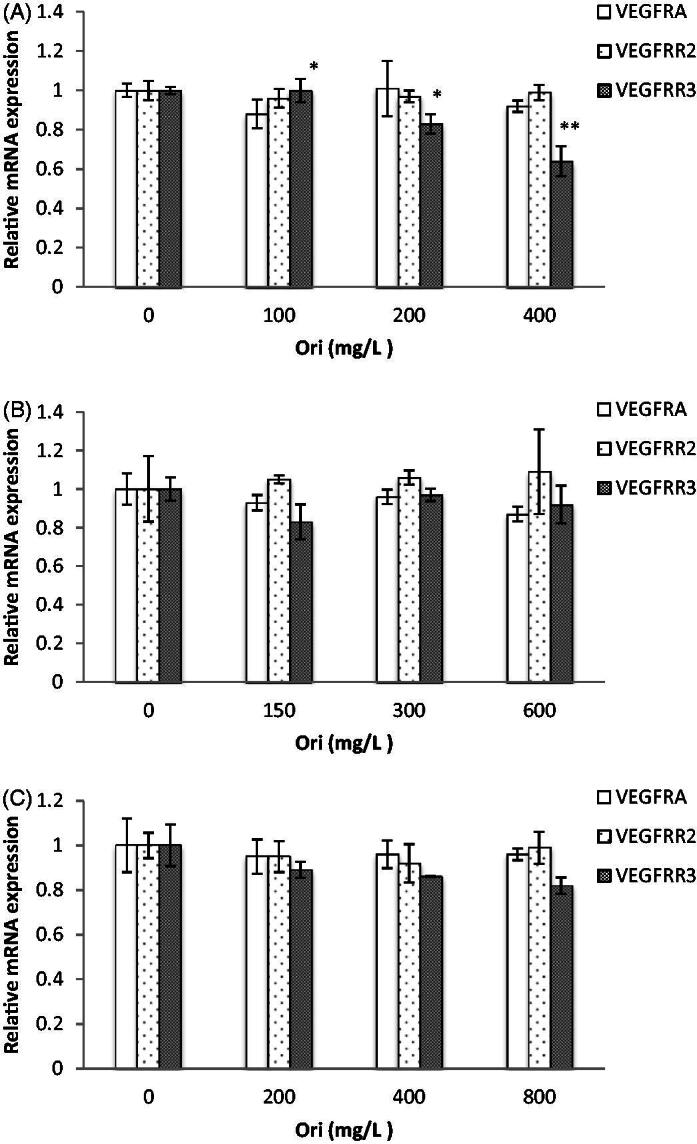
Comparison of gene expression between experiment groups. Note: Asterisks indicate statistically significant differences between different concentration of oridonin groups and the without oridonin group (*p < 0.05; **p < 0.01). Each error bar represents the standard deviations of at least three experiments. A: embryos; B: larvae; C: adult. Ori: Oridonin.

Zebrafish embryos treated with oridonin (100, 200 and 400 mg/L) showed decreased mRNA expression of *VEGFR3* (Figure 7(A)), showing significant differences compared to that in the control (*p* < 0.05, *p* < 0.05 and *p* < 0.01, respectively). However, the expression of *VEGFA* and *VEGFR2* did not differ significantly between any of the oridonin groups and their respective controls. More importantly, the results showed that oridonin had no effect on the mRNA expression of *VEGFA*, *VEGFR2* and *VEGFR3* in larval zebrafish and the adult zebrafish (Figures 7(B,C)).

## Discussion

In recent years, oridonin has attracted researchers’ attention because of its antitumor effect, and related studies had clarified that the mechanism of oridonin mainly depends on the c-Met, Notch and VEGF signalling pathways. Furthermore, studies on selenium nanoparticles and chemical structure optimization both have made progress in indicating that oridonin is a candidate chemotherapeutic agent cancer (Ding et al. 2013; Pi et al. 2017). However, the safety study of oridonin *in vivo* is still unclear.

Zebrafish have become an important tool in high-throughput drug screening because it is affordable for assessing the toxicity and efficacy of novel drugs, allowing a high predictive power on possible human drug-induced liabilities. Overall, in a fast and cost-effective manner, zebrafish models (embryos, larvae and adult models) can bridge the gap between preclinical in vitro safety assays and rodent models (Cornet et al. 2017; Kulkarni et al. 2017; Chen et al. 2017). Our previous research showed that oridonin inhibits tumour growth and metastasis, mainly through its antiangiogenic effects. This study aimed to determine whether oridonin affects zebrafish at different developmental stages. In the present study, we investigated oridonin safety in embryo, larval and adult zebrafish models and evaluated the developmental effects of oridonin at different end-points and exposure periods. We showed that oridonin exerted developmental effects on zebrafish embryos. Oridonin exhibited a two-phase effect: at low doses, it promoted spontaneous movements and hatching rates, but at high doses, it inhibited these parameters. Oridonin reduced the number of heartbeats, compared to that in the control group (*p* < 0.01). Oridonin induced abnormalities in zebrafish, such as uninflated swim bladder and pericardial congestion, at an EC_50_ of 411.94 mg/L. Oridonin decreased body length and increased swimming speed, compared to those observed in the control group (both *p* < 0.01). However, oridonin had no effect on the larval and adult models.

Zebrafish embryos at 6 hpf are considered suitable as tools for evaluating the embryonic toxicity of chemical drugs by observing abnormal phenotypes during embryonic development (Zhang et al. 2015). Therefore, this study exposed zebrafish embryos to oridonin from 6 hpf. Rhythmic spontaneous bending of the tail is the first movement of a zebrafish that originates from innervation of spinal neurons, and this movement is independent of higher brain inputs (Saint-Amant and Drapeau 1998). The first movements in zebrafish embryos at 28.5 °C were observed at 17 hpf because of the presence of functional neurons adjoining the somite (Fraysse et al. 2006). The peak of spontaneous movements is observed at 21 hpf, and a delay of 4 h may be observed at lower temperatures. In the present study, spontaneous movements per 20 s per embryo were recorded for 24, 27 and 30 hpf. After exposure to oridonin until 30 hpf, spontaneous movements first increased and then decreased with increasing concentrations of oridonin. Compounds that induce neurotoxicity in humans cause similar neurotoxicity in zebrafish, thereby confirming zebrafish as a predictive model for assessing neurotoxicity (Parng et al. 2007). As observed in previous studies, spontaneous movements resulted from the uncontrolled action potential of motor neurons. Some terpenoids, such as ginkgolides, ginsenosides and cannabinoids, are potential agents against Alzheimer’s disease because of their promising *in vitro* and *in vivo* biological activities (Yoo and Park 2012). Previous studies have suggested the potential therapeutic application of diterpenoids for neurodegenerative disorders (Dao et al. 2010). Oridonin, a natural diterpenoid compound, has shown anti-neuroinflammatory and neuroregulatory effects in several *in vitro* studies (Liu et al. 2007; Chen et al. 2012). Oridonin ameliorates neuropathological changes and behavioural deficits in a mouse model of cerebral amyloidosis, suggesting that oridonin is a promising therapeutic option for neurodegenerative diseases (Zhang et al. 2013). Oridonin might be a promising candidate treatment for Alzheimer disease (Xu et al. 2009; Wang et al. 2014, 2016).

Generally available antiangiogenic agents are known to have serious side effects. For example, hypertension is a well-known systemic adverse effect of treatment with VEGF inhibitors (Antonuzzo et al. 2017; Randrup Hansen et al. 2017). Our previous research showed that oridonin had antiangiogenic effects *in vitro* and *in vivo* (Tian et al. 2017). In the present study, we investigated the development of zebrafish heart because zebrafish offers several distinct advantages as a genetic and embryonic model system to study cardiovascular disease (Bakkers 2011). The development of the zebrafish heart is a complex process that includes cell proliferation, migration and differentiation, and the heart occurs earliest in the development of zebrafish embryo, with the heart rate steady at 48 hpf. This study detected the heart rate of zebrafish embryos at 48, 51 and 54 hpf, and the results showed that oridonin obviously decreased heart rate in a dose-dependent manner in the tested concentration range, but showed no obvious abnormalities in heart tissue, thereby showing that oridonin as relatively safe with respect to hypertension. The zebrafish model will be developed as a very powerful model to study cardiac development, with improved availability for basic researchers interested in studying cardiac diseases (Mickoleit et al. 2014).

Similar to the mammalian lungs, the zebrafish swim bladder arises from an outgrowth of the foregut endoderm and is in close temporal and spatial proximity to the liver and pancreas (Spooner and Wessells 1970; Field et al. 2003). Exposure to oridonin induced the occurrence of uninflated swim bladder at 120 hpf. Oridonin increased larval swimming speed and decreased larval body length; moreover, oridonin stimulated hatching rate at low concentrations, but inhibited hatching rate at high concentrations.

Phenotypic changes in angiogenesis are always involved in angiogenesis-related signalling pathways. VEGF is a potent proangiogenic factor that stimulates endothelial cell proliferation, migration and tube formation, which are key events of the angiogenic process (Rousseau et al. 2000). Moreover, VEGF also plays a role in normal physiological functions, such as haematopoiesis and development (Ferrara et al. 2003). The previous study showed that VEGFA signalling directly affects myocardium fusion, indicating that it is the initial cause of heart defects. Moreover, VEGFA regulates heart morphogenesis in a dose-dependent manner (Zhu et al. 2017). Experiments in zebrafish showed that changes in the levels of VEGFR3 modulate aortic lumen diameter, consistent with flow-dependent remodelling, thus showing its relevance to vessel remodelling *in vivo* (Baeyens et al. 2015). Notably, this study found that the downregulation of *VEGFR3* gene expression may be related to the occurrence of abnormalities following oridonin exposure during embryonic development. Taken together, the present study showed that oridonin showed adverse effects on the development of zebrafish embryos by downregulating *VEGFR3* gene expression; thus, this is the first study to report the side effects of oridonin *in vivo*.

## Conclusions

This study showed the adverse effects of oridonin at the early stages of zebrafish development, which is a crucial finding regarding the safety of oridonin for therapy. However, it was clearly indicated that oridonin application was relatively safe and potential as an effective drug candidate for angiogenesis-related diseases.
